# Finite-resource teleportation stretching for continuous-variable systems

**DOI:** 10.1038/s41598-018-33332-y

**Published:** 2018-10-15

**Authors:** Riccardo Laurenza, Samuel L. Braunstein, Stefano Pirandola

**Affiliations:** Computer Science and York Centre for Quantum Technologies, University of York, York, YO10 5GH United Kingdom

## Abstract

We show how adaptive protocols of quantum and private communication through bosonic Gaussian channels can be simplifed into much easier block versions that involve resource states with finite energy. This is achieved by combining an adaptive-to-block reduction technique devised earlier, based on teleportation stretching and relative entropy of entanglement, with a recent finite-resource simulation of Gaussian channels. In this way, we derive weak converse upper bounds for the secret-key capacity of phase-insensitive Gaussian channels which approximate the optimal limit for infinite energy. Our results apply to both point-to-point and repeater-assisted private communications.

## Introduction

Establishing the ultimate limits of quantum and private communications is important^1,2^, not only to explore the boundary of quantum mechanics but also to provide benchmarks for testing the practical performance of experimental and technological implementations. This problem is important for quantum systems of any dimension^3,4^ and, in particular, for infinite-dimensional ones, also known as continuous-variable (CV) systems^5–8^. In quantum information and quantum optics, the most important CV systems are the bosonic modes of the electromagnetic field^6^, which are typically used at the optical or telecom wavelengths. In any protocol of quantum communication, such modes are subject to loss and noise, and the most typical and basic model for such kind of decoherence is the single-mode Gaussian channel.

It is known that protocols of private communication and quantum key distribution (QKD) are limited in both rate and distance due to decoherence, no matter if the communication line is a free-space link or a fiber connection. This limitation is perhaps best simplified by the rate-loss scaling of ideal single-photon BB84 protocol^9^ whose optimal rate scales as *η*/2 secret bits per channel use, where *η* is the transmissivity of the channel. Recently, this fundamental rate-loss limit has been fully characterized. By optimizing over the most general key-generation protocols, Pirandola-Laurenza-Ottaviani-Banchi^10^ have established the secret-key capacity of the lossy channel to be $K(\eta )=-\,{\mathrm{log}}_{{\rm{2}}}\mathrm{(1}-\eta )$, which is about 1.44*η* secret bits per channel use at long distances ($\eta \simeq 0$). This result sets a general benchmark for quantum repeaters^11–24^ and completes a long-standing investigation started back in 2009^25,26^, when the best known lower bound was discovered.

The main technique that led to establishing the previous capacity is based on a suitable combination of two ingredients, the relative entropy of entanglement (REE)^27–29^ suitably extended from states to channels (using results from refs^30–32^), and teleportation stretching, which reduces any adaptive (feedback-assisted) quantum protocol over an arbitrary channel into a much simpler block version. This latter technique is a full extension and generalization of previous approaches^33–35^ that only worked for specific classes of channels and were designed to reduce quantum error correcting code protocols into entanglement distillation. Without doubts, the generalization to an arbitrary task over an arbitrary quantum channel has been one of the key insights of ref.^10^, and this has been widely exploited in recent literature, with a number of follow-up papers in the area of quantum Shannon theory^4^, e.g., on strong converse rates, broadcast capacities, etc. See ref.^36^ for a recent review on these topics and refs^36,37^ for rigorous proofs of some related claims.

The core of teleportation stretching is the idea of channel simulation, where an arbitrary quantum channel is replaced by local operations and classical communication (LOCC) applied to the input and a suitable resource state^10^. This powerful idea is rooted in the protocol of teleportation^38,39^ and first proposed in ref.^33^, despite originally limited to the simulation of Pauli channels^40^ (see also ref.^41^). Later, this core idea was extended to generalized teleportation protocols^35,42^ and CV teleportation^43^ in refs^34,44^. The final and more general form involves a simulation via arbitrary LOCCs, as formulated in ref.^10^. In particular, the simulation of bosonic channels is typically asymptotic, which means that they need a suitable limit over sequences of resource states, which comes from the fact that the Choi matrices of such channels are asymptotic states^10^. Most importantly, such a simulation needs a careful control of the simulation error as first considered in ref.^10^, otherwise technical divergences may appear in the results. This crucial aspect is discussed in detail in ref.^36^, which also provides a direct comparison of the various simulation techniques appeared in the literature.

Here we consider a different type of simulation for bosonic Gaussian channels, which is based on finite-energy two-mode Gaussian states as recently introduced in ref.^45^. We use this particular simulation at the core of teleportation stretching in order to simplify adaptive protocols. This not only represents an interesting design (with potential applications beyond this work) but also allows us to derive upper bounds for the secret-key capacities of phase-insensitive Gaussian channels which approximate well the asymptotic results of ref.^10^.

## Results

### Preliminaries on the simulation of bosonic channels

As discussed in ref.^10^ an arbitrary quantum channel ${\mathcal E}$ can be simulated by a trace-preserving LOCC ${\mathscr{T}}$ and a suitable resource state *σ*, i.e.

1 $$ {\mathcal E} (\rho )={\mathscr{T}}\,(\rho \otimes \sigma \mathrm{).}$$

A channel is called *σ*-stretchable if it has *σ* as a resource state via some LOCC simulation as in Eq. (1). An important case is when the channel is Choi-stretchable, which means that the resource state can be chosen to be its Choi matrix $\sigma ={\rho }_{ {\mathcal E} }:\,=I\otimes  {\mathcal E} ({\rm{\Phi }})$, with Φ being a maximally entangled state. For a bosonic channel, the maximally entangled state is an Einstein-Podolsky-Rosen (EPR) state with infinite energy, so that the Choi matrix of a bosonic channel is energy-unbounded. For this reason one has to work with a sequence of two-mode squeezed vacuum states^5^ Φ^*μ*^ with variance $\mu =\bar{n}+\mathrm{1/2}$, where $\bar{n}$ is the average number of thermal photons in each mode. By definition, the EPR state is defined as ${\rm{\Phi }}:=\mathop{\mathrm{lim}}\limits_{\mu }\,{{\rm{\Phi }}}^{\mu }$ and the Choi matrix of a bosonic channel ${\mathcal E}$ is defined by

2 $${\rho }_{ {\mathcal E} }:=\mathop{\mathrm{lim}}\limits_{\mu }\,{\rho }_{ {\mathcal E} }^{\mu },\,{\rho }_{ {\mathcal E} }^{\mu }= {\mathcal I} \otimes  {\mathcal E} ({{\rm{\Phi }}}^{\mu }\mathrm{).}$$

This means that the simulation needs to be asymptotic, i.e., of the type

3 $$ {\mathcal E} (\rho )=\mathop{\mathrm{lim}}\limits_{\mu }\,{\mathscr{T}}\,(\rho \otimes {\rho }_{ {\mathcal E} }^{\mu }\mathrm{).}$$

(More generally, one also needs to consider sequences of LOCCs ${{\mathscr{T}}}^{\mu }$, so that the asymptotic simulation reads ${\mathcal E} (\rho )={\mathrm{lim}}_{\mu }{{\mathscr{T}}}^{\mu }(\rho \otimes {\rho }_{ {\mathcal E} }^{\mu })$. For simplicity we omit this technicality, referring the reader to ref.^10^ for more details.)

In ref.^10^, we identified a simple sufficient condition for a quantum channel to be Choi-stretchable, even asymptotically as in Eq. (3): teleportation covariance. In the bosonic case, a channel ${\mathcal E}$ is teleportation-covariant if, for any random displacement *D* (as induced by CV teleportation)^39,43^, we may write

4 $$ {\mathcal E} (D\rho {D}^{\dagger })=V {\mathcal E} (\rho ){V}^{\dagger },$$

for some unitary *V*. It is clear that bosonic Gaussian channels are teleportation covariant and, therefore, Choi-stretchable, with asymptotic simulation as in Eq. (3).

### Simulation of Gaussian channels with finite-energy resource states

Recently, ref.^45^ proposed a variant of Gaussian channel simulation^10^, where single-mode phase-insensitive Gaussian channels are simulated by applying CV teleportation to a particular class of finite-energy Gaussian states as the resource. Consider a single-mode Gaussian state with mean value $\bar{x}$ and covariance matrix (CM) **V**^5^. The action of a single-mode Gaussian channel can be expressed in terms of the statistical moments as

5 $$\bar{x}\to {\bf{T}}\bar{x},\,{\bf{V}}\to {\bf{T}}{\bf{V}}{{\bf{T}}}^{T}+{\bf{N}},$$

where **T** and ${\bf{N}}={{\bf{N}}}^{T}$ are 2 × 2 real matrices satisfying suitable conditions^5^. In particular, the channel is called phase-insensitive if these two matrices take the specific diagonal forms

6 $${\bf{T}}=\sqrt{\eta }{\bf{I}},\,{\bf{N}}=\nu {\bf{I}}$$

where $\eta \in {\mathbb{R}}$ is a transmissivity parameter, while $\nu \ge 0$ represents added noise.

According to ref.^45^, a phase-insensitive Gaussian channel ${ {\mathcal E} }_{\eta ,\nu }$ can be simulated as follows

7 $${ {\mathcal E} }_{\eta ,\nu }(\rho )={{\mathscr{T}}}_{\eta }(\rho \otimes {\sigma }_{\nu }),$$

where ${{\mathscr{T}}}_{\eta }$ is the Braunstein-Kimble protocol with gain $\sqrt{\eta }$ ^43,46^, and *σ*_*v*_ is a zero-mean two-mode Gaussian state with CM

8 $${\bf{V}}({\sigma }_{\nu })=(\begin{array}{cc}a{\bf{I}} & c{\bf{Z}}\\ c{\bf{Z}} & b{\bf{I}}\end{array}),$$

where^45^

9 $$a=\frac{2b+(\eta -\mathrm{1)}{e}^{-2r}}{2\eta },\,\,c=\frac{2b-{e}^{-2r}}{2\sqrt{\eta }},$$

10 $$b=\frac{-\,|\eta -1|+\eta {e}^{2r}+{e}^{-2r}}{\mathrm{2[}-\,{e}^{2r}|\eta -1|+\eta +\mathrm{1]}},$$

and the entanglement parameter *r* ≥ 0 is connected to the channel parameter via the relation

11 $$\nu =\frac{{e}^{-2r}}{2}(\eta +\mathrm{1).}$$

Note that, with respect to the fomulas of ref.^45^, we have an extra 1/2 factor in Eqs (8) and (11). This is due to the different notation we adopt here. We set the quadrature variance of the vacuum state to be 1/2, while it was equal to 1 in ref.^45^. Also note that, in the simulation of Eq. (7), one uses a Braunstein-Kimble protocol with an ideal CV Bell detection. The latter is an asymptotic measurement defined in the limit of infinite squeezing, i.e., infinite energy. For this reason, the finite-energy aspect of the simulation in Eq. (7) only refers to the resource state.

### Finite-resource teleportation stretching of an adaptive protocol

Here we plug the previous finite-resource simulation into the tool of teleportation stretching. We start by providing some necessary definitions on adaptive protocols and secret-key capacity. Then, we review a general upper bound (weak converse) based on the REE. Finally, following the recipe of refs^10,47^. we show how to use the finite-resource simulation to simplify an adaptive protocol and reduce the REE bound to a single-letter quantity.

### Adaptive protocols and secret-key capacity

The most general protocol for key generation is based on adaptive LOCCs, i.e., local operations assisted by unlimited and two-way classical communication. Each transmission through the quantum channel is interleaved by two of such LOCCs. The general formalism can be found in ref.^10^ and goes as follows. Assume that two remote users, Alice and Bob, have two local registers of quantum systems (modes), **a** and **b**, which are in some fundamental state ${\rho }_{{\bf{a}}}\otimes {\rho }_{{\bf{b}}}$. The two parties applies an adaptive LOCC Λ_0_ before the first transmission.

In the first use of the channel, Alice picks a mode *a*_1_ from her register **a** and sends it through the channel ${\mathcal E}$. Bob gets the output mode *b*_1_ which is included in his local register **b**. The parties apply another adaptive LOCC Λ_1_. Then, there is the second transmission and so on. After *n* uses, we have a sequence of LOCCs $\{{{\rm{\Lambda }}}_{0},{{\rm{\Lambda }}}_{1},\,\ldots ,\,{{\rm{\Lambda }}}_{n}\}$ characterizing the protocol ${\mathcal L}$ and an output state ${\rho }_{{\bf{a}}{\bf{b}}}^{n}$ which is *ε*-close to a target private state^48^ with *nR*_*n*_ bits. Taking the limit of large *n* and optimizing over the protocols, we define the secret-key capacity of the channel

12 $$K( {\mathcal E} )=\mathop{{\rm{\sup }}}\limits_{ {\mathcal L} }\,\mathop{\mathrm{lim}}\limits_{n}\,{R}_{n}\mathrm{.}$$

### General upper bound

According to Theorem 1 (weak converse) in ref.^10^, a general upper bound for $K( {\mathcal E} )$ is given in terms of the REE of the output state ${\rho }_{{\bf{a}}{\bf{b}}}^{n}$

13 $$K( {\mathcal E} )\le {E}_{R}^{\ast }( {\mathcal E} ):=\mathop{{\rm{\sup }}}\limits_{ {\mathcal L} }\,\mathop{\mathrm{lim}}\limits_{n}\frac{{E}_{R}({\rho }_{{\bf{a}}{\bf{b}}}^{n})}{n}\mathrm{.}$$

Recall that the REE of a state *ρ* is defined as ${E}_{R}(\rho )={{\rm{\inf }}}_{{\sigma }_{{\rm{sep}}}}S(\rho ||{\sigma }_{{\rm{sep}}})$, where *σ*_sep_ is a separable state and the relative entropy is defined by $S(\rho ||{\sigma }_{{\rm{sep}}}):={\rm{Tr}}[\rho ({\mathrm{log}}_{{\rm{2}}}\,\rho -{\mathrm{log}}_{2}{\sigma }_{{\rm{sep}}})]$. These definitions can be easily adapted for asymptotic states of bosonic systems.

Note that the first and simplest proof of Eq. (13) can be found in ref.^49^ (the second arxiv version of ref.^10^). To avoid potential misunderstandings or misinterpretations of this proof, we report here the main points. For any protocol whose output ${\rho }_{{\bf{a}}{\bf{b}}}^{n}$ is *ε*-close (in trace norm) to target private state with rate *R*_*n*_ and dimension *d*, we may write

14 $$n{R}_{n}\le {E}_{R}({\rho }_{{\bf{a}}{\bf{b}}}^{n})+4\varepsilon \,{\mathrm{log}}_{2}\,d+2{H}_{2}(\varepsilon ),$$

where *H*_2_ is the binary Shannon entropy. For distribution through a discrete variable (DV) channel, whose output is a DV state, we may write

15 $${\mathrm{log}}_{2}\,d\le \alpha n{R}_{n},$$

for some constant *α* [see also Eq. (21) of ref.^49^]. The exponential scaling in Eq. (15) comes from previous results in refs^31,32^. The latter showed that, for any adaptive protocol with rate *R*_*n*_, there is another protocol with the same asymptotic rate while having an exponential scaling for *d*.

The extension to a CV channel is achieved by a standard argument of truncation of the output Hilbert space. After the last LOCC Λ_*n*_, Alice and Bob apply a truncation LOCC ${{\mathbb{T}}}_{d}$ which maps the output state ${\rho }_{{\bf{a}}{\bf{b}}}^{n}$ into a truncated version ${\rho }_{{\bf{a}}{\bf{b}}}^{n,d}={{\mathbb{T}}}_{d}({\rho }_{{\bf{a}}{\bf{b}}}^{n})$ with total dimension *d*. The total protocol ${{\mathbb{T}}}_{d}\,\circ \, {\mathcal L} =\{{{\rm{\Lambda }}}_{0},\,{{\rm{\Lambda }}}_{1},\,\cdots ,\,{{\rm{\Lambda }}}_{n},\,{{\mathbb{T}}}_{d}\}$ generates an output that is *ε*-close to a DV private state with *nR*_*n,d*_ bits. Therefore, we may directly re-write Eq. (14) as

16 $$n{R}_{n,d}\le {E}_{R}({\rho }_{{\bf{a}}{\bf{b}}}^{n,d})+4\varepsilon \,{\mathrm{log}}_{2}\,d+2{H}_{2}(\varepsilon \mathrm{).}$$

Both the output and the target are DV states, so that we may again write Eq. (15). In fact, since the Hilbert space is finite-dimensional, the proof of refs^31,32^ automatically applies, i.e., the protocol can be stopped after *n*_0_ uses, and then repeated *m* times in an i.i.d. fashion, with *n* = *n*_0_*m*. Key distillation applied to the *m* DV output copies implies a number of bits of CCs which is linear in *m* which, in turn, leads to an exponential scaling of *d* in *n*.

Because ${{\mathbb{T}}}_{d}$ is a trace-preserving LOCC, we exploit the monotonicity of the REE ${E}_{R}({\rho }_{{\bf{a}}{\bf{b}}}^{n,d})\le {E}_{R}({\rho }_{{\bf{a}}{\bf{b}}}^{n})$ and rewrite Eq. (16) as

17 $${R}_{n,d}\le \frac{{E}_{R}({\rho }_{{\bf{a}}{\bf{b}}}^{n})+2{H}_{2}(\varepsilon )}{n\mathrm{(1}-4\alpha \varepsilon )}\mathrm{.}$$

Taking the limit for large *n* and small *ε* (weak converse), this leads to

18 $$\mathop{\mathrm{lim}}\limits_{n}\,{R}_{n,d}\le \mathop{\mathrm{lim}}\limits_{n}\,{n}^{-1}{E}_{R}({\rho }_{{\bf{a}}{\bf{b}}}^{n}\mathrm{).}$$

The crucial observation is that in the right-hand side of the latter expression, there is no longer dependence on the truncation *d*. Therefore, in the optimization of *R*_*n,d*_ over all protocols ${{\mathbb{T}}}_{d}\,\circ \, {\mathcal L}$ we can implicitly remove the truncation. Pedantically, we may write

19 $$K({\mathscr{E}})=\mathop{sup}\limits_{d}\,\mathop{sup}\limits_{{{\mathbb{T}}}_{d}\circ {\mathscr{L}}}\,\mathop{{\rm{l}}{\rm{i}}{\rm{m}}}\limits_{n}\,{R}_{n,d}\le \mathop{sup}\limits_{{\mathscr{L}}}\,\mathop{{\rm{l}}{\rm{i}}{\rm{m}}}\limits_{n}\,{n}^{-1}{E}_{R}({\rho }_{{\bf{a}}{\bf{b}}}^{n}):={E}_{R}^{\ast }({\mathscr{E}}).$$

#### **Remark 1**

*Note that the truncation argument was explicitly used in* ref.^49^ *to extend the bound to CV channels. See discussion after* Eq. (23) *of* ref.^49^. *There a cut-off was introduced for the total CV Hilbert space at the output. Under this cutoff, the derivation for DV systems was repeated, finding an upper bound which does not depend on the truncated dimension (this was done by using the monotonicity of the REE exactly as here). The cutoff was then relaxed in the final expression as above. The published version*^10^ *includes other equivalent proofs but they have been just given for completeness*.

### Simplification via teleportation stretching

One of the key insights of ref.^10^ has been the simplification of the general bound in Eq. (13) to a single-letter quantity. For bosonic Gaussian channels, this was achieved by using teleportation stretching with asymptotic simulations, where a channel is reproduced by CV teleportation over a sequence of Choi-approximating resource states. Here we repeat the procedure but we adopt the finite-resource simulation of ref.^45^. Recall that, differently from previous approaches^33–35^, teleportation stretching does not reduce a protocol into entanglement distillation but maintains the task of the original protocol, so that adaptive key generation is reduced to block (non-adaptive) key generation. See ref.^36^ for comparisons and clarifications.

Assume that the adaptive protocol is performed over a phase-insensitive Gaussian channel ${ {\mathcal E} }_{\eta ,\nu }$, so that we may use the simulation in Eq. (7), where ${{\mathscr{T}}}_{\eta }$ is the Braunstein-Kimble protocol with gain $\sqrt{\eta }$ and *σ*_*ν*_ is a zero-mean two-mode Gaussian state, specified by Eqs (8–11). We may re-organize an adaptive protocol in such a way that each transmission through ${ {\mathcal E} }_{\eta ,\nu }$ is replaced by its resource state *σ*_*ν*_. At the same time, each teleportation-LOCC ${{\mathscr{T}}}_{\eta }$ is included in the adaptive LOCCs of the protocol, which are all collapsed into a single LOCC ${\bar{{\rm{\Lambda }}}}_{\eta }$ (trace-preserving after averaging over all measurements). In this way, we may decompose the output state ${\rho }_{{\bf{a}}{\bf{b}}}^{n}:\,={\rho }_{{\bf{a}}{\bf{b}}}({ {\mathcal E} }_{\eta ,\nu }^{\otimes n})$ as

20 $${\rho }_{{\bf{a}}{\bf{b}}}^{n}={\bar{{\rm{\Lambda }}}}_{\eta }({\sigma }_{\nu }^{\otimes n}\mathrm{).}$$

The computation of ${E}_{R}({\rho }_{{\bf{a}}{\bf{b}}}^{n})$ can now be remarkably simplified. In fact, we may write

21 $${E}_{R}({\rho }_{{\bf{a}}{\bf{b}}}^{n})=\mathop{{\rm{\inf }}}\limits_{{\sigma }_{{\rm{sep}}}}\,S({\rho }_{{\bf{a}}{\bf{b}}}^{n}||{\sigma }_{{\rm{sep}}})\mathop{\le }\limits^{\mathrm{(1)}}\mathop{{\rm{\inf }}}\limits_{{\sigma }_{{\rm{sep}}}}\,S[{\bar{{\rm{\Lambda }}}}_{\eta }({\sigma }_{\nu }^{\otimes n})||{\bar{{\rm{\Lambda }}}}_{\eta }({\sigma }_{{\rm{sep}}})]\mathop{\le }\limits^{\mathrm{(2)}}\mathop{{\rm{\inf }}\,}\limits_{{\sigma }_{{\rm{sep}}}}S({\sigma }_{\nu }^{\otimes n}||{\sigma }_{{\rm{sep}}})={E}_{R}({\sigma }_{\nu }^{\otimes n}),$$

where: (1) we consider the fact that ${\bar{{\rm{\Lambda }}}}_{\eta }({\sigma }_{{\rm{sep}}})$ form a subset of specific separable states, and (2) we use the monotonicity of the relative entropy under the trace-preserving LOCC ${\bar{{\rm{\Lambda }}}}_{\eta }$. Therefore, by replacing in Eq. (13), we get rid of the optimization over the protocol (disappearing with ${\bar{{\rm{\Lambda }}}}_{\eta }$) and we may write

22 $$K({ {\mathcal E} }_{\eta ,\nu })\le \mathop{\mathrm{lim}}\limits_{n}\frac{{E}_{R}({\sigma }_{\nu }^{\otimes n})}{n}:\,={E}_{R}^{\infty }({\sigma }_{\nu })\le {E}_{R}({\sigma }_{\nu }),$$

where we use the fact that the regularized REE is less than or equal to the REE. Thus, we may write the following theorem:

#### **Theorem 2**

*Consider a phase-insensitive bosonic Gaussian channel* ${ {\mathcal E} }_{\eta ,\nu }$, *which is stretchable into a two-mode Gaussian state σ*_*ν*_ *as given in Eqs* (8–11). *Its secret-key capacity must satisfy the bound*

23 $$K({ {\mathcal E} }_{\eta ,\nu })\le {E}_{R}({\sigma }_{\nu }):\,=\mathop{{\rm{\inf }}}\limits_{{\sigma }_{{\rm{sep}}}}\,S({\sigma }_{\nu }||{\sigma }_{{\rm{sep}}}\mathrm{).}$$

Note that the new bound in Eq. (23) cannot beat the asymptotic bound established by ref.^10^ for bosonic channels, i.e.,

24 $$K({ {\mathcal E} }_{\eta ,\nu })\le \mathop{{\rm{\inf }}}\limits_{{\sigma }_{{\rm{sep}}}^{\mu }}\,\mathop{\mathrm{lim}\,{\rm{\inf }}}\limits_{\mu \to +\infty }\,S({\rho }_{{ {\mathcal E} }_{\eta ,\nu }}^{\mu }||{\sigma }_{{\rm{sep}}}^{\mu }),$$

where ${\rho }_{{ {\mathcal E} }_{\eta ,\nu }}^{\mu }$ is a Choi-approximating sequence as in Eq. (2), and ${\sigma }_{{\rm{sep}}}^{\mu }$ is an arbitrary sequence of separable states converging in trace norm. This can be seen from a quite simple argument^50^. In fact, according to Eqs (2) and (7), we may write

25 $${\rho }_{{ {\mathcal E} }_{\eta ,\nu }}^{\mu }= {\mathcal I} \otimes { {\mathcal E} }_{\eta ,\nu }({{\rm{\Phi }}}^{\mu })= {\mathcal I} \otimes {{\mathscr{T}}}_{\eta }({{\rm{\Phi }}}^{\mu }\otimes {\sigma }_{\nu })={\rm{\Delta }}({\sigma }_{\nu }),$$

where Δ is a trace-preserving LOCC. Therefore, ${E}_{R}({\rho }_{{ {\mathcal E} }_{\eta ,\nu }}^{\mu })\le {E}_{R}({\sigma }_{\nu })$ and this relation is inherited by the bounds above. Notwithstanding this *no go* for the finite-resource simulation, we show that its performance is good and reasonably approximates the infinite-energy bounds that are found via Eq. (24).

### Finite-resource bounds for phase insensitive Gaussian channels

We now proceed by computing the REE in Eq. (23) for the class of single-mode phase-insensitive Gaussian channels. For this, we exploit the closed formula for the quantum relative entropy between Gaussian states which has been derived in ref.^10^ by using the Gibbs representation for Gaussian states^51^. Given two Gaussian states ${\rho }_{1}({u}_{1},\,{V}_{1})$ and ${\rho }_{2}({u}_{2},\,{V}_{2})$, with respective statistical moments *u*_*i*_ and *V*_*i*_, their relative entropy is

26 $$S({\rho }_{1}||{\rho }_{2})=-\,{\rm{\Sigma }}({V}_{1},\,{V}_{1})+{\rm{\Sigma }}({V}_{1},\,{V}_{2}),$$

where we have defined

27 $${\rm{\Sigma }}({V}_{1},\,{V}_{2}):\,=\frac{\mathrm{ln}\,{\rm{\det }}({V}_{2}+\frac{i{\rm{\Omega }}}{2})+{\rm{Tr}}({V}_{1}{G}_{2})+{\delta }^{T}{G}_{2}\delta }{2\,\mathrm{ln}\,2}$$

with $\delta ={u}_{1}-{u}_{2}$ and ${G}_{2}=2i{\rm{\Omega }}\,{\coth }^{-1}\mathrm{(2}i{V}_{2}{\rm{\Omega }})$ ^51^, where the matrix Ω is the symplectic form.

The computation of the REE involves an optimization over the set of separable states. Following the recipe of ref.^10^ we may construct a good candidate directly starting from the CM in Eq. (8). This separable state has CM with the same diagonal blocks as in Eq. (8), but where the off-diagonal term is replaced as follows

28 $$c\to {c}_{{\rm{sep}}}:\,=\sqrt{(a-\mathrm{1/2)(}b-\mathrm{1/2)}}\mathrm{.}$$

By using this separable state ${\tilde{\sigma }}_{{\rm{sep}}}$ we may write the further upper bound

29 $${E}_{R}({\sigma }_{\nu })\le {\rm{\Psi }}( {\mathcal E} ):\,=S({\sigma }_{\nu }||{\tilde{\sigma }}_{{\rm{sep}}}\mathrm{).}$$

In the following, we compute this bound for the various types of phase-insensitive Gaussian channels.

### Thermal-loss channel

This channel can be modelled as a beam splitter of transmissivity *η* where the input signals are combined with a thermal environment such that the quadratures transform according to $\hat{{\bf{x}}}\to \sqrt{\eta }\hat{{\bf{x}}}+\sqrt{1-\eta }{\hat{{\bf{x}}}}_{th}$, where ${\hat{{\bf{x}}}}_{th}$ is in a thermal state with $\bar{n}$ photons. In terms of the statistical moments, the action of the thermal-loss channel ${ {\mathcal E} }_{\eta ,\bar{n}}$ can be described by the matrices in Eq. (6) with parameter $\nu =\mathrm{(1}-\eta )(\bar{n}+\mathrm{1/2)}$. This means that the squeezing parameter *r* of the resource state now reads

30 $$r=\frac{1}{2}\,\mathrm{ln}[\frac{\eta +1}{(2\bar{n}+1)\mathrm{(1}-\eta )}]\mathrm{.}$$

**Figure 1:**
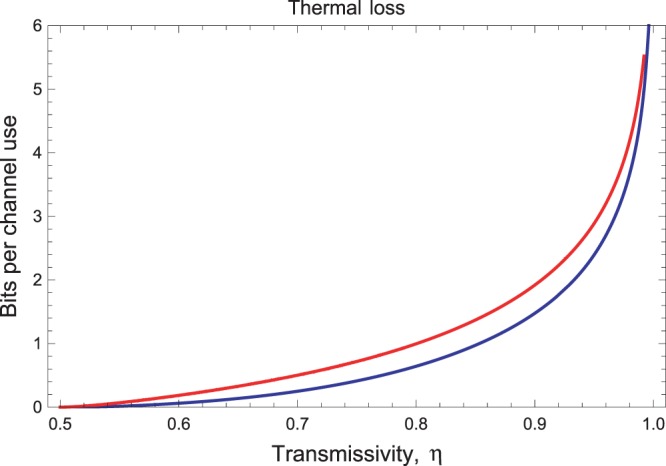
Finite-resource bound ${\rm{\Psi }}({ {\mathcal E} }_{\eta ,\bar{n}})$ on the secret-key capacity of the thermal loss channel (red upper curve) as a function of the transmissivity *η*, compared with the infinite-energy bound ${\rm{\Phi }}({ {\mathcal E} }_{\eta ,\bar{n}})$ (blue lower curve) derived in ref.^10^. The curves are plotted for $\bar{n}=1$ thermal photons.

By combining this relation with the ones in Eq. (10) and computing the relative entropy, we find the finite-resource bound ${\rm{\Psi }}({ {\mathcal E} }_{\eta ,\bar{n}})$ which is plotted in Fig. 1 and therein compared with the infinite-energy bound ${\rm{\Phi }}({ {\mathcal E} }_{\eta ,\bar{n}})$ derived in ref.^10^. The latter is given by^10^

31 $${\rm{\Phi }}({ {\mathcal E} }_{\eta ,\bar{n}})=-\,{\mathrm{log}}_{2}\mathrm{[(1}-\eta ){\eta }^{\bar{n}}]-h(\bar{n}),$$

for $\bar{n} < \eta \mathrm{/(1}-\eta )$ and zero otherwise, and we set $h(x):\,=(x+\mathrm{1)}\,{\mathrm{log}}_{2}(x+\mathrm{1)}-x\,{\mathrm{log}}_{2}\,x$. It is clear that we have

32 $$K({ {\mathcal E} }_{\eta ,\bar{n}})\le {\rm{\Phi }}({ {\mathcal E} }_{\eta ,\bar{n}})\le {\rm{\Psi }}({ {\mathcal E} }_{\eta ,\bar{n}}),$$

but the two upper bounds are reasonably close.

### Noisy amplifier channel

**Figure 2:**
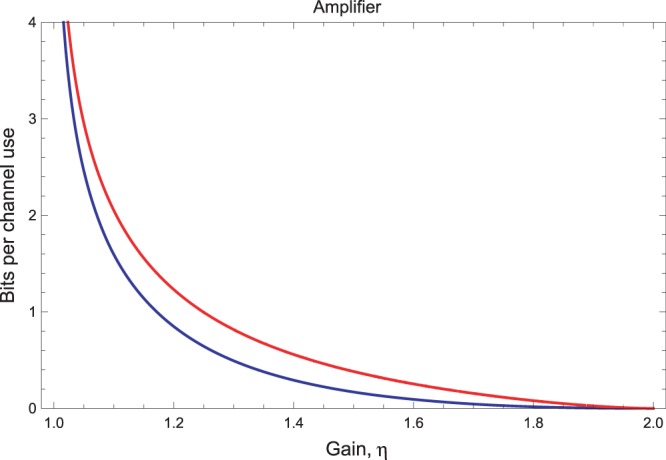
Finite-resource bound ${\rm{\Psi }}({ {\mathcal E} }_{\eta ,\bar{n}})$ on the secret-key capacity of the noisy amplifier channel (red upper curve) as a function of the gain *η*, compared with the optimal bound for infinite energy ${\rm{\Phi }}({ {\mathcal E} }_{\eta ,\bar{n}})$ (blue lower curve). The two curves are plotted for $\bar{n}=1$ thermal photons.

A noisy quantum amplifier is described by $\hat{{\bf{x}}}\to \sqrt{\eta }\hat{{\bf{x}}}+\sqrt{\eta -1}{\hat{{\bf{x}}}}_{th}$, where *η* > 1 is the gain and ${\hat{{\bf{x}}}}_{th}$ is in a thermal state with $\bar{n}$ photons. This channel ${ {\mathcal E} }_{\eta ,\bar{n}}$ is described by the matrices in Eq. (6) with parameter $\nu =(\eta -\mathrm{1)(}\bar{n}+\mathrm{1/2)}$. By repeating the previous calculations, we find the finite-resource bound ${\rm{\Psi }}({ {\mathcal E} }_{\eta ,\bar{n}})$ plotted in Fig. 2 and where it is compared with the infinite-energy bound^10^

33 $${\rm{\Phi }}({ {\mathcal E} }_{\eta ,\bar{n}})={\mathrm{log}}_{2}(\frac{{\eta }^{\bar{n}+1}}{\eta -1})-h(\bar{n}),$$

for $\bar{n} < {(\eta -\mathrm{1)}}^{-1}$ and zero otherwise.

### Additive-noise Gaussian channel

**Figure 3:**
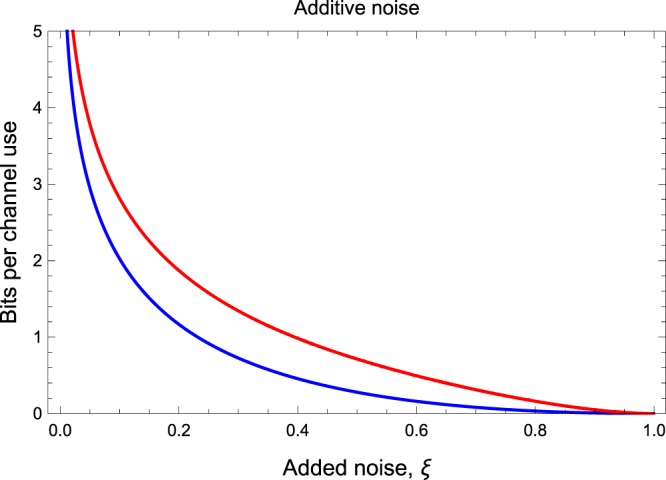
Finite-resource bound ${\rm{\Psi }}({ {\mathcal E} }_{\xi })$ on the secret-key capacity of the additive noise Gaussian channel (red upper curve) as a function of the added noise *ξ*, compared with the optimal bound for infinite energy ${\rm{\Phi }}({ {\mathcal E} }_{\xi })$ (blue lower curve).

Another important channel is represented by the additive-noise Gaussian channel, which is the simplest model of bosonic decoherence. In terms of the input-output transformations, the quadratures transforms according to $\hat{{\bf{x}}}\to \hat{{\bf{x}}}+{(z,z)}^{T}$ where *z* is a classical Gaussian variable with zero mean and variance *ξ* ≥ 0. This channel ${ {\mathcal E} }_{\xi }$ is described by the matrices in Eq. (6) with *η* = 1 and *ν* = *ξ*. The finite-resource bound ${\rm{\Psi }}({ {\mathcal E} }_{\xi })$ on the secret key capacity is plotted in Fig. 3 and compared with the infinite-energy bound^10^

34 $${\rm{\Phi }}({ {\mathcal E} }_{\xi })=\frac{\xi -1}{\mathrm{ln}\,2}-{\mathrm{log}}_{2}\,\xi ,$$

for *ξ* < 1, while zero otherwise.

### Pure-loss channel

For the pure-loss channel, the upper bound derived in the limit of infinite energy^10^ coincides with the lower bound computed with the reverse coherent information^25,26^. This means that we are able to fully characterize the secret-key capacity for this specific bosonic channel. This is also known as the Pirandola-Laurenza-Ottaviani-Banchi (PLOB) bound^10^

35 $${\mathscr{K}}(\eta )=-\,{\mathrm{log}}_{2}\mathrm{(1}-\eta )\simeq 1.44\eta \,{\rm{for}}\,\eta \simeq \mathrm{0,}$$

and fully characterizes the fundamental rate-loss scaling of point-to-point quantum optical communications.

Consider now the finite-resource teleportation simulation of a pure-loss channel. It is easy to check that we cannot use the parametrization in Eq. (10). In fact, for a pure-loss channel, we have $\nu =\mathrm{(1}-\eta \mathrm{)/2}$ so that Eq. (11) provides ${e}^{2r}=\mathrm{(1}+\eta \mathrm{)/(1}-\eta )$. Replacing the latter in Eq. (10), we easily see that we have divergences (e.g., the denominator of *b* becomes zero). For the pure loss channel, we therefore use a different simulation, where the resource state is a two-mode squeezed state with CM^50^

36 $${\sigma }_{\eta }=(\begin{array}{cc}a{\bf{I}} & \sqrt{{a}^{2}-\mathrm{1/4}}{\bf{Z}}\\ \sqrt{{a}^{2}-\mathrm{1/4}}{\bf{Z}} & a{\bf{I}}\end{array}),\,a=\frac{\eta +1}{\mathrm{2(1}-\eta )}\mathrm{.}$$

**Figure 4:**
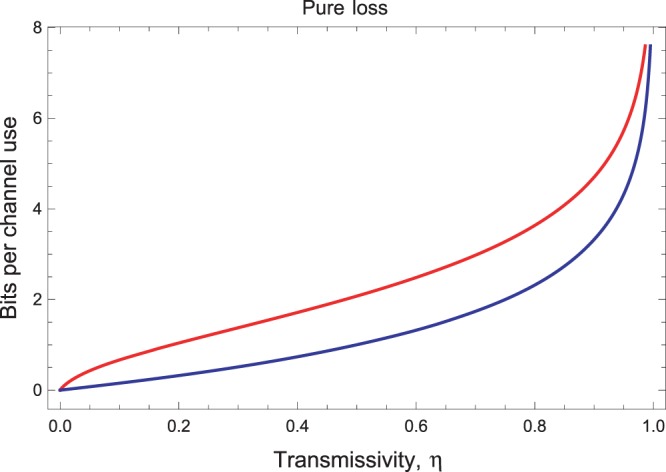
Finite-resource bound ${\rm{\Psi }}({ {\mathcal E} }_{\eta })$ on the secret-key capacity of the pure-loss channel (red upper curve) as a function of the transmissivity *η*, compared with its secret key capacity or PLOB bound $K(\eta )=-\,{\mathrm{log}}_{2}\mathrm{(1}-\eta )$ (blue lower curve).

By exploiting this resource state, we derive the bound ${\rm{\Psi }}({ {\mathcal E} }_{\eta })$ shown in Fig. 4, where it is compared with the secret-key capacity *K*(*η*).

### Extension to repeater-assisted private communication

Here we extend the previous treatment to repeater-assisted private communication. We consider the basic scenario where Alice **a** and Bob **b** are connected by a chain of *N* quantum repeaters $\{{{\bf{r}}}_{1},\,\ldots ,\,{{\bf{r}}}_{N}\}$, so that there are a total of *N* + 1 quantum channels $\{{ {\mathcal E} }_{i}\}$ between them. Assume that these are phase-insensitive Gaussian channels ${ {\mathcal E} }_{i}:\,={ {\mathcal E} }_{{\eta }_{i},{\nu }_{i}}$ with parameters $({\eta }_{i},{\nu }_{i})$. The most general adaptive protocol for key distribution through the chain is described in ref.^52^ and goes as follows.

Alice, Bob and all the repeaters prepare their local registers $\{{\bf{a}},{{\bf{r}}}_{1},\ldots ,{{\bf{r}}}_{N},{\bf{b}}\}$ into a global initial state *ρ*^0^ by means of a network LOCC Λ_0_, where each node in the chain applies LOs assisted by unlimited and two-way CCs with all the other nodes. In the first transmission, Alice picks a system ${a}_{1}\in {\bf{a}}$ and sends it to the first repeater; after another network LOCC Λ_1_, the first repeater communicates with the second repeater; then there is another network LOCC Λ_2_ and so on, until Bob is eventually reached, which terminates the first use of the chain.

After *n* uses of the chain, we have a sequence of network LOCCs ${\mathcal L}$ defining the protocol and an output state ${\rho }_{{\bf{a}}{\bf{b}}}^{n}$ for Alice and Bob which approximates some target private state with *nR*_*n*_ bits. By taking the limit for large *n* and optimizing over the protocols, we define the end-to-end or repeater-assisted secret-key capacity^52^

37 $$K(\{{{\mathscr{E}}}_{i}\})=\mathop{{\rm{s}}{\rm{u}}{\rm{p}}}\limits_{{\mathscr{L}}}\,\mathop{{\rm{l}}{\rm{i}}{\rm{m}}}\limits_{n}\,{R}_{n}.$$

As shown in ref.^52^, we may extend the upper bound of Eq. (13). Then, we may use teleportation stretching and optimize over cuts of the chain, to simplify the bound to a single-letter quantity.

**Figure 5:**
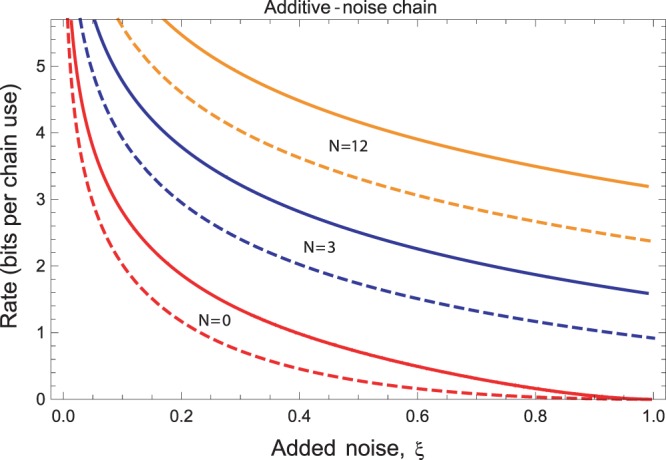
Secret-key capacity of a chain of *N* equidistant repeaters creating *N* + 1 additive-noise Gaussian channels with variances ${\xi }_{i}=\xi /(N+\mathrm{1)}$. We compare the finite-resource upper bound ${\rm{\Psi }}(\{{ {\mathcal E} }_{i}\})$ (solid lines) with the infinite-energy upper bound ${\rm{\Phi }}(\{{ {\mathcal E} }_{i}\})$ (dashed lines) for different values of *N* as a function of the overall added noise of the chain *ξ*.

The network-reduction technique of ref.^52^ can be implemented by using the specific finite-resource simulation of Eq. (7), which leads to the following possible decompositions of the output state

38 $${\rho }_{{\bf{a}}{\bf{b}}}^{n}={\bar{{\rm{\Lambda }}}}_{i}({\sigma }_{{\nu }_{i}}^{\otimes n}),\,{\rm{for}}\,{\rm{any}}\,i=\mathrm{1,}\,\ldots ,\,N,$$

where ${\bar{{\rm{\Lambda }}}}_{i}$ is a trace-preserving LOCC and ${\sigma }_{{\nu }_{i}}$ is the resource state associated with the *i*th Gaussian channel. By repeating the derivation of ref.^52^, this leads to

39 $$K(\{{ {\mathcal E} }_{i}\})\le \mathop{{\rm{\min }}}\limits_{i}\,{E}_{R}({\sigma }_{{\nu }_{i}})\le \mathop{{\rm{\min }}}\limits_{i}\,S({\sigma }_{{\nu }_{i}}||{\tilde{\sigma }}_{i,{\rm{sep}}}):\,={\rm{\Psi }}(\{{ {\mathcal E} }_{i}\mathrm{\})\ ,}$$

where Ψ is the upper bound coming from our choice of the separable state ${\tilde{\sigma }}_{i,{\rm{sep}}}$ in the REE. This upper bound needs to be compared with the one ${\rm{\Phi }}(\{{ {\mathcal E} }_{i}\})$ obtained in the limit of infinite energy^52^. As an example, consider an additive-noise Gaussian channel with noise variance *ξ*. Let us split the communication line by using *N* “equidistant” repeaters, in such a way that each link is an additive-noise Gaussian channel ${ {\mathcal E} }_{i}$ with the same variance ${\xi }_{i}=\xi /(N+\mathrm{1)}$. It is easy to check that this is the optimal configuration for the repeaters. From Eq. (39), we derive ${\rm{\Psi }}(\{{ {\mathcal E} }_{i}\})={\rm{\Psi }}({ {\mathcal E} }_{\xi /(N+\mathrm{1)}})$. This bound is plotted in Fig. 5 where we can se an acceptable approximation of the corresponding infinite-energy bound ${\rm{\Phi }}(\{{ {\mathcal E} }_{i}\})$.

## Discussion

In this work we have presented a design for the technique of teleportation stretching^10^ for single-mode bosonic Gaussian channels, where the core channel simulation^45^ is based on a finite-energy two-mode Gaussian state processed by the Braunstein-Kimble protocol^43^ with suitable gains. Such an approach removes the need for using an asymptotic simulation where the sequence of states approximates the energy-unbounded Choi matrix of a Gaussian channel, even though the infinite energy limit remains at the level of Alice’s quantum measurement which is ideally a CV Bell detection (i.e., a projection onto displaced EPR states). Using this approach we compute the weak converse bound for the secret key capacity of all phase-insensitive single-mode Gaussian channels, which include the thermal-loss channel, the quantum amplifier and the additive-noise Gaussian channel. We find that the bounds so derived are reasonably close to the tightest known bound established in ref.^10^ by using asymptotic Choi matrices. We have considered not only for point-to-point communication but also a repeater-assisted scenario where Alice and Bob are connected by a chain of quantum repeaters.

The tools developed here may have other applications. For instance, they may be applied to multi-point protocols^53^ and more complex quantum networks^52^. In an arbitrary multi-hop quantum communication network, the end-to-end capacities under single- and multi-path routing strategies may be expressed in terms of the REE of finite-energy resource states. In particular, these states can be identified by solving classical problems of network information theory (widest path or maximum flow) following the same approach in ref.^52^. In the context of quantum metrology, a finite-resource simulation (different from the one employed in the present paper) has been recently exploited in ref.^54^. The stretching strategy adopted therein allows one to simplify the most general adaptive protocol for quantum parameter estimation into a block scheme, so that one can write an upper bound for the quantum Fisher information in terms of a finite-energy resource state. This allows one to lower-bound the minimum variance of the error that affects the adaptive estimation of noise parameters in Gaussian channels, with good approximation of the optimal bounds established in ref.^55^ but based on asymptotic Choi matrices.

### Note added

Our work first appeared on the arXiv in June 2017^56^. It has been revised after an imprecision in ref.^45^ was fixed in ref.^57^. Independently and simultaneously, a related work^58^ also built on the techniques of ref.^10^, but its claims were restricted to a point-to-point thermal-loss channel in the non-asymptotic scenario.

## Data Availability

The datasets generated during the current study are available from the corresponding author on reasonable request.
